# Dynamic Transcriptome-Based Weighted Gene Co-expression Network Analysis Reveals Key Modules and Hub Genes Associated With the Structure and Nutrient Formation of Endosperm for Wax Corn

**DOI:** 10.3389/fpls.2022.915400

**Published:** 2022-06-09

**Authors:** Tingchun Li, Fanna Kong, Qing Dong, Dafeng Xu, Guihu Liu, Yanli Lei, Huaying Yang, Yingbing Zhou, Cheng Li

**Affiliations:** Tobacco Research Institute, Anhui Academy of Agricultural Sciences, Hefei, China

**Keywords:** waxy corn, floury endosperm, dynamic comparative transcriptome analysis, weighted gene co-expression network analysis, modules and hub genes

## Abstract

The endosperm of corn kernel consists of two components, a horny endosperm, and a floury endosperm. In the experiment, a kind of floury endosperm corn was identified. The result of phenotypic trait analysis and determination of amino acid content showed that the floury endosperm filled with the small, loose, and scattered irregular spherical shape starch granules and contained higher content of amino acid. The starch biochemical properties are similar between floury corns and regular flint corn. By using dynamically comparative transcriptome analysis of endosperm at 20, 25, and 30 DAP, a total of 113.42 million raw reads and 50.508 thousand genes were obtained. By using the weighted gene co-expression network analysis, 806 genes and six modules were identified. And the turquoise module with 459 genes was proved to be the key module closely related to the floury endosperm formation. Nine zein genes in turquoise module, including two *zein-alpha A20* (*Zm00001d019155* and *Zm00001d019156*), two *zein-alpha A30* (*Zm00001d048849* and *Zm00001d048850*), one *50 kDa gamma-zein* (*Zm00001d020591*), one *22 kDa alpha-zein 14* (*Zm00001d048817*), one *zein-alpha 19D1* (*Zm00001d030855*), one *zein-alpha 19B1* (*Zm00001d048848*), and one *FLOURY 2* (*Zm00001d048808*) were identified closely related the floury endosperm formation. Both *zein-alpha 19B1* (*Zm00001d048848*) and *zein-alpha A30* (*Zm00001d048850*) function as source genes with the highest expression level in floury endosperm. These results may provide the supplementary molecular mechanism of structure and nutrient formation for the floury endosperm of maize.

## Introduction

Corn is one of the most important grain crops in the world. The corn endosperm mainly contains primarily starch surrounded by a protein matrix, accounting for 83% of the corn kernel. Ordinary corn kernel endosperm comprises soft and vitreous starch (Rasper and Walker, 2000; Xu et al., 2019). Based on the quality and quantity of both types, corn grains could be divided into the dent, flint, flour, sweet, and popcorn (Gwirtz and Garcia-Casal, 2014).

Generally, the starch granules are embedded compactly in the protein membrane in the vitreous endosperm (Kikuchi et al., 1982). But in the floury endosperm, the starch granules are arranged loosely (Kikuchi et al., 1982). Most recent research demonstrated that the hardness of endosperm was closely related to the protein’s composition. Three types of proteins were isolated in the endosperm, including albumin-globulins, prolamins (zeins in corn seeds), and true glutelins (Landry and Moureaux, 1980). The three kinds of proteins, respectively, accounted for 13, 48, and 35% of total nitrogen in the floury endosperm, and 4, 79, and 15% of that in the vitreous endosperm, which implied that the hardness of the seed was closely associated with zein protein (Landry et al., 2004). In the corn endosperm, four types of zein proteins were identified based on the protein structure (Wu and Messing, 2012). The M_r_ 19- and 22-kD proteins were accounted for 75–85% of zein proteins, which are called alpha-zeins. The M_r_ 15-kD protein is the beta-zein, which makes up 10–15% of the whole zein proteins. The M_r_ 50-, 27-, and 16-kD proteins constitute 5–10% of the name of gamma-zeins, and the M_r_ 18- and 10-kD proteins are called the delta-zein with a small proportion in the endosperm (Wu and Messing, 2012). The previous researcher found that hard endosperm contains more α-zeins fractions than soft endosperm fractions (Dombrink-Kurtzman and Bietz, 1993). On the contrary, there are nearly twice γ-zeins in the soft endosperm than in the hard endosperm (Dombrink-Kurtzman and Bietz, 1993). Maize endosperm hardness is determined by the zein proteins (Robutti et al., 1997). In addition, further study on the relationship between starch compositions and hardness of endosperm revealed that the starch granules from soft endosperm had a rough appearance with randomly distributed pores on their surface (Dombrink-Kurtzman and Knutson, 1997). But few pores were detectable on granules from the hard endosperm. And the soft endosperm has lower amylose content than the hard endosperm. Otherwise, the genes related to the zeins biosynthesis have been identified. By using cDNA libraries from developing endosperm of the B73 maize inbred line, 15 different, complete coding sequences were identified and grouped into the α-, γ-, and δ-zein gene subfamilies (Woo et al., 2001). Of these genes, five genes belong to the 19-kD α-zein transcript, four genes encode 22-kD α-zein protein, four genes annotated as 27-kD γ-zein biosynthesis genes, and two genes encode the δ-zein protein. Furthermore, constructing an overlapping cosmid library of *Zea mays* BSSS53 further isolated and sequenced all 23 members of the 22-kD α-zein gene family (Song et al., 2001). Twenty-two of them were mapped on chromosome 4S. The gene expression analysis revealed that only seven of these genes appear active with different transcriptional regulations based on the maize cDNA databases (Song et al., 2001). Moreover, to obtain the target gene-related nutrient and structure formation of endosperm, maize mutants were usually used as the samples with the obvious phenotypic changes. Several genes, including *fl1*, *fl2*, *fl3*, and *fl4*, were cloned and demonstrated to play a key role caused the formation of floury endosperm (Coleman et al., 1997; Holding et al., 2007; Wang et al., 2014, 2018). Among those genes, *fl1* encodes a novel endoplasmic reticulum protein which plays a vital role in the regulation of zein protein body formation, *fl2* functions as a 22-kD α-zein with a defective signal peptide, *fl3* annotated as a member of a novel class of plant-specific zinc-dependent DNA-binding proteins ZmPLATZ12, and *fl*4 encodes a 19-kD α-zein z1A subfamily member (Coleman et al., 1997; Holding et al., 2007; Wang et al., 2014, 2018). Otherwise, maize opaque endosperm mutants commonly exhibit floury kernels too. Most studies showed that the opaque endosperm contained a soft texture with increased nutritional quality (Zhang et al., 2018). For example, the gene *o1* encodes a myosin XI motor protein; *o2* encodes a bZIP transcription factor (Wang et al., 2012; Lappe et al., 2018).

An inbred wax corn line with floury endosperm was isolated in this work. The physiological and biochemical indexes of endosperm were analyzed. Using dynamic comparative transcriptomic analysis combined with weighted gene co-expression network analysis (WGCNA), their key modules and hub genes related to the hardness and starch synthesis were analyzed between the floury endosperm and the vitreous endosperm. These results will provide valuable genetic resources for further studies of floury corn and contribute to an expanding view on the physiologic change and its relationship with transcriptomic variation, which may give more insight into the gene expression profiles related to starch and protein synthesis in the corn seeds.

## Materials and Methods

### Plant Material

A floury endosperm corn seed was firstly isolated from an F2 segregating population of the cross recombination inbred line W024 and inbred line W133 in the farm of Tobacco Research Institute, Anhui province, People’s Republic of China. After successive self-pollination at F2, F3, F4, and F5 generations, a stable F6 generation was obtained. This study selected a floury endosperm corn inbred line W056 and a flint inbred line W042 from the F7 generation for the downstream experiment. The inbred lines were cultivated with regular water and fertilizer management on the farm of Tobacco Research Institute in the year 2019. The endosperm was collected and frozen immediately in liquid nitrogen at 20, 25, and 30 days after pollination (DAP). Then the samples were stored at −70°C for comparative transcriptome analysis. The analysis of phenotypic trait and biochemical properties were conducted with the help of Shanghai Sanshu Biotechnology Co., Ltd. (Shanghai, China).

### Microscopic Observation of Floury Endosperm

The mature seeds were collected and dissected with a scalpel and tweezers. Then, the cross-section profiles were observed and documented by using the anatomical lens (Olympus SZ5161, Japan).

### Scanning of Starch Granule Morphology

For scanning electron microscopy (SEM) analysis of Starch granule morphology, mature maize endosperm was transversely cut with a razor blade and mounted on a metal stub. After sputter-coated with gold, the samples were observed and photographed with a Zeiss Merlin Compact scanning electron microscope (SEM, Zeiss, Oberkochen, Germany).

### Determination of Physiological Traits

The mature seed was ground into powder to extract starch following physiological analysis. To determine the crystallinity and branching degree of the starch, 20 g powder was soaked in sodium pyrosulfite aqueous solution containing 10 mg/g alkaline protease at 42°C for 24 h. After filtration, the residue was collected and standing overnight. The supernatant was removed until the starch became white, and then it was dried naturally. After being balanced for a week in an incubator, the samples were ground and filtered for analysis using an X’Pert Pro X-ray diffractometer (PANalytical, Netherlands). The Nal scintillation counter was used to determine the X-ray strength at the scanning range from 5° to 60°. The DS-SS-RS was designed as 1, −1, −0.1 mm.

### The Molecular Weight Distribution of Starch

At first, 5 mg starch samples were dissolved in 1 ml DMSO at 100°C overnight. Then, 3 ml absolute ethanol was added to the solution. After centrifugation, the residue was dried and dissolved in 3 ml 0.1 mol NaNO_3_ at 121°C for 20 min. The supernatant was collected for the following experiment after centrifugation. The molecular weight distribution was analyzed using GPC-RI-MALS methods. In detail, the RI dictator (Optilab T-rEX, Wyatt Technology, CA, United States) equipped with mals (DAWN HELEOS II, Wyatt Technology, CA, United States) and Series 1500 Pump (Waters) was employed using 0.1 mol NaNO_3_ as flow phase with 0.4 ml/min at 60°C. Three columns Ohpak SB-805 HQ (300 × 8 mm), Ohpak SB-804 HQ (300 × 8 mm) and Ohpak SB-803 HQ (300 × 8 mm), were series-connected to obtain the results.

### The Thermal Properties of the Starch

The starch sample with 10 mg was balanced in 30 μL distilled water for 24 h at room temperature and sealed in an alumina crucible. The differential scanning calorimetry (DSC, Q2000, TA Instruments, United States) was used to analyze the enthalpy change of the samples with 10°C min speed from 30 to 95°C.

### Amino Acid Content Determination

The dried powder with 50 mg was dissolved in 1 ml 6 mol hydrochloric acid for 24 h. The supernatant was collected after centrifugation and dried at 80°C in an oven. Then it was dissolved in 800 μl 0.1 mol hydrochloric acid and incubated in a shaker at 37°C for 1 h. The supernatant was collected by centrifugation at 12,000 rpm for 10 min. The UPLC was equipped with a chromatographic column (ACCQ-TAG TMULTRA C18, 1.7 um, 2.1 × 100 mm). The AccQ⋅TagtmUltra Eluent A Concentrate and AccQ⋅TagtmUltra Eluent B were selected as mobile phases. The flow rate was designed as 0.7 ml/min at 50°C. Twenty-four kinds of amino acid components, including L-Threonine, L-Alanine, L-Proline, L-Cystine, L-Lysine monohydrochloride, L-Tyrosine, L-Methionine, L-Valine, L-Isoleucine, L-Leucine, L-Phenylalanine, L-Tryptophan, L-Cysteine hydrochloride, rans-4-Hydroxy-L-proline, γ-Aminobutyric acid and (R)-(-)-2-Aminobutyric acid from Waters (United States) were selected as standards.

### Total RNA Extraction

Total RNA was extracted with RNAprep Pure Plant Kit (Tiangen, China) using 200 mg of endosperm tissue. Its quality and concentration were determined using NanoDrop 2000 (Thermo, United States). The RNAs were independently collected three times, creating three biological replicates.

### cDNA Library Construction and Sequencing

The cDNA library construction and Illumina sequencing were carried out on an Illumina Hiseq 2500 platform with a 200 bp paired-end read at Beijing Novogene Biological Information Technology Co., Ltd. (Beijing, China)^1^.

### Data Filtering and Assembly

The high-quality clean reads were obtained after removing adaptor sequences, duplicated sequences, ploy-N (reads with unknown nucleotides), and low-quality reads. Then the cleaned sequences were mapped to the maize B73 reference genome (AGPv4)^2^ (Schnable et al., 2009). The final transcriptome assembly was conducted using the TopHat 2 tool as previously described by Mortazavi et al. (2008) and Kim et al. (2013). The fragments per kilobase million mapped reads (FPKM) were calculated according to the method described by Trapnell et al. (2010). All raw data were deposited in the GenBank Short Read Archive^3^ and could be accessible at the NCBI SRA database with the accession number PRJNA820289.

### Weighted Gene Co-expression Network Analysis

To obtain the target genes related to the structure and nutrition formation of floury endosperm, the R package (R 4.1.2) was downloaded from the website https://cran.r-projet.org/bin/windows/base/ and installed to run the following analysis. In detail, all genes were selected firstly. After removing genes with a low FPKM (FPKM < 1), the left genes were used to calculate every gene’s variance between different samples using the variance function. Then, the top 32% of genes with a maximum level of variance were screened out using quantile function. The WGCNA analysis was conducted according to the method as described by Langfelder and Horvath (2008). The pick-Soft Threshold function was conducted to confirm the appropriate power value to run the weighted gene co-expression network analysis. Then the Pearson’s correlations among all genes were calculated to create the adjacency matrix and form a topological matrix. The one-step network construction and module detection were used to identify the gene modules. The gene groups are represented with different colors to distinguish each module from the others. The module eigengenes were calculated to merge the close modules with the value of MEDissThres = 0.2. Eigengene dendrogram and Eigengene adjacency heatmap were plotted to differentiate the expression pattern of modules.

To obtain key module correlated with floury endosperm, the floury endosperm trait of W056 was set as “1,” the normal endosperm trait of W042 was designated to be “0.” The *P* value was calculated to evaluate the correlation of module-trait relationships. The values of intramodular connectivity, gene significance, and kME were used to identify the hub genes. The gene networks were displayed using Cytoscape v.3.9.1. The Gene Ontology (GO) annotation and KEGG pathways analysis of the candidate genes were conducted using the NovoMagic online tools^4^.

## Results

### Identification of Floury Endosperm Corn and Measurement of Its Starch Composition

**FIGURE 1:**
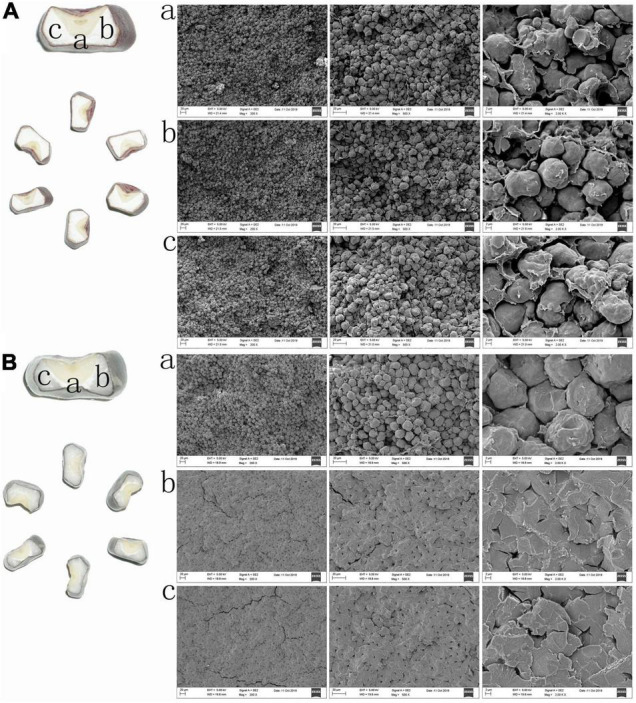
The difference of starch structure for endosperm of W042 and W056.

As is shown in Figure 1, the endosperm of inbred line W056 exhibited an entire floury surface after the cross-section of the mature seed. Otherwise, the endosperm of inbred line W042 appeared to have a floury central core surrounded by a vitreous endosperm layer for the mature seed. SEM revealed that the floury endosperm was filled with the small, loose and scattered irregular spherical shape starch granules for inbred line W056. Contrarily, the endosperm of W042 was composed of two different parts. In the central core, the starch granules were the bigger irregular spherical shape particles. But in the surrounding central core, all starch granules were large, polyhedral, and tightly packed blocks.

### Crystallinity and Branching Degree

The XRD was used as an effective method to determine the crystal structure of starch. As is shown in Table 1, both samples displayed typical A-type diffraction patterns with the same diffraction peaks at the 2θ value, suggesting that phenotype changes did not alter the polymorphic structure type of the starch.

**TABLE 1:** The crystal structure analysis of starch for W042 and W056.

Sample name	Crystal structure type	Diffraction peaks at 2θ value (angle)							
		5°	15°	17°	19°	19.5°	22.0°	23°	24°
W042	A type		√	√	√		√		
W056	A type		√	√	√		√		

### Starch Granule and Molecular Weight

**FIGURE 2:**
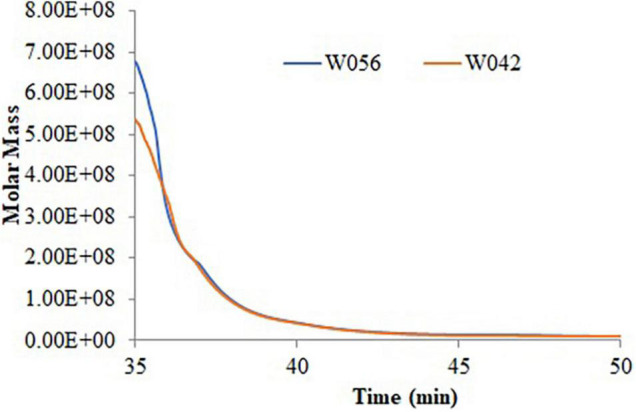
The molecular weight distribution of the starch for the endosperm of W042 and W056.

Table 2 and Figure 2 exhibited the information on the molecular weight of starch. Compared with W042, W056 displayed higher Mp, Mw, and Mz values. But the value of Mn was lower.

**TABLE 2:** The starch granule and molecular weight for W042 and W056.

Sample name	Mn (kDa)	Mp (kDa)	Mw (kDa)	Mz (kDa)	Polydispersity	
					Mw/Mn	Mz/Mn
W042	27839.2 (±2.1%)	205550.1 (±6.2%)	103790.6 (±6.3%)	250733.0 (±17.4%)	3.728 (±6.6%)	9.006 (±14.9%)
W056	26986.0 (±2.9%)	209295.3 (±12.5%)	112845.4 (±10.1%)	310957.3 (±26.8%)	4.182 (±10.5%)	11.523 (±22.4%)

Polydispersity indicated the broadness of the molecular weight distribution, which was calculated as the ratio of Mw/Mn and Mz/Mn. The lower value of polydispersity explained a narrower molecular weight distribution of starch. This study showed that both the Mw/Mn and Mz/Mn values were higher in W056, which showed a broader molecular weight distribution of floury amylopectin.

### The Thermal Properties of the Starch

Table 3 displays the thermal characteristics of the starch according to the results of the DSC analysis. The values of To, Tp, and Tc were higher in W056. But its value of ΔH was lower. The results indicated that a higher temperature was required to disrupt the double-helical order for the starch of W056. The lower value of ΔH suggested a reduction in the crystallinity degree of starch by decreasing the double-helical order content, which is in accord with the result of the phenotypic analysis.

**TABLE 3:** The thermal properties of the starch for W042 and W056.

Sample name	To	Tp	Tc	ΔH
W042	65.73 ± 0.17b	70.41 ± 0.10b	75.89 ± 0.18b	13.76 ± 0.24a
W056	67.14 ± 0.76a	72.04 ± 0.18a	77.34 ± 0.07a	13.56 ± 0.34a

*The lower case letters indicated the significant difference with P < 0.05.*

### The Content of Amino Acid

**FIGURE 3:**
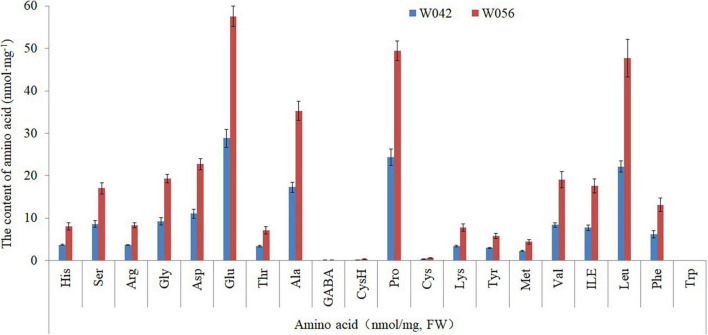
The contents of different amino acid components for W042 and W056.

As shown in Figure 3, the amino acid content was determined. The result showed that a total of 20 components were identified. The contents of Glu, Ala, Pro, and Len were higher if compared with other components, including Asp, Gly, Val, and so on. Between two corns inbred lines, the amino acid content was higher in the endosperm of W056 than that of W042.

### Summary of Transcriptome Data

The endosperms at 20, 25, and 30 DAP development stages were collected to construct RNA-seq libraries with three biological replications. As a result, a total of 113.42 million raw reads were obtained (Table 4). After filtration of low-quality reads, 115.54 million clean reads (approximately 167.31 Gb clean bases) were isolated with a 9.295 Gb for each sample. Over 91.21% of the Q30 values and no less than 52.71% of GC contents were concluded. The average value of Q30 and GC contents were 92.38 and 54.44%, respectively. A total of 88.28–92.29% of the clean reads were successfully matched to the maize reference genome. The ratios of uniquely mapped reads were between 88.28 and 92.26%. These results implied the reliability of the transcriptome data.

**TABLE 4:** Summary of six separately pooled RNA sequencing results.

Sample name	Raw reads (bp)	Clean reads (bp)	Clean bases	Q20 (%)	Q30 (%)	GC content (%)	Uniquely mapped	Total mapped
W56_20_1	69841582	69020950	10.35G	97.66	93.15	55.18	58595307 (84.89%)	63173266 (91.53%)
W56_20_2	62911702	61438136	9.22G	97.03	92.17	54.58	51230146 (83.38%)	55246619 (89.92%)
W56_20_3	57558104	56413570	8.46G	97.05	92.21	54.25	46968913 (83.26%)	50678109 (89.83%)
W56_25_1	73676038	72869982	10.93G	97.69	93.32	53.45	60916299 (83.6%)	66822466 (91.7%)
W56_25_2	59078918	57961508	8.69G	96.86	91.83	52.8	47380711 (81.75%)	52243732 (90.14%)
W56_25_3	65862564	64414240	9.66G	96.98	92.01	52.72	52154901 (80.97%)	57934108 (89.94%)
W56_30_1	68578136	67828792	10.17G	97.78	93.48	53.6	57431451 (84.67%)	62577626 (92.26%)
W56_30_2	51492516	50579832	7.59G	96.55	91.21	52.74	41490885 (82.03%)	45382407 (89.72%)
W56_30_3	56988358	55846494	8.38G	96.94	92.01	52.71	46219043 (82.76%)	50589610 (90.59%)
W42_20_1	63164396	62350912	9.35G	97.69	93.3	55.93	53218365 (85.35%)	56235051 (90.19%)
W42_20_2	55986022	54947704	8.24G	97.06	92.26	55.07	45948132 (83.62%)	48682188 (88.6%)
W42_20_3	60731496	59225076	8.88G	97.2	92.54	54.92	49393616 (83.4%)	52336439 (88.37%)
W42_25_1	61771952	60977452	9.15G	97.54	92.99	55.98	51894662 (85.1%)	54944476 (90.11%)
W42_25_2	82173306	80861182	12.13G	96.9	91.92	54.97	67662956 (83.68%)	72078984 (89.14%)
W42_25_3	60097974	59132592	8.87G	96.83	91.7	55.1	49660871 (83.98%)	52775697 (89.25%)
W42_30_1	65238660	64381258	9.66G	97.56	92.94	55.56	54743189 (85.03%)	57980220 (90.06%)
W42_30_2	61473346	60388946	9.06G	96.95	91.99	55.15	50225116 (83.17%)	53313555 (88.28%)
W42_30_3	57581100	56814248	8.52G	96.91	91.97	55.23	47206225 (83.09%)	50215987 (88.39%)

Among all transcriptome data, 50.508 thousand genes were identified and almost unequally distributed on chromosomes 1–10 (Supplementary Table 1). To obtain the target genes related to the formation of floury endosperm, the R package was used to isolate candidate genes. Firstly, we removed the genes with a low FPKM (FPKM < 1), and 20,131 genes were left. Then, the R package was used to calculate the variances for every gene between different samples. At last, the top 32% of genes with a maximum level of variance were identified. Finally, a total of 806 candidate genes were obtained for further analysis.

### Gene Ontology Annotation and KEGG Pathways Analysis of Candidate Genes

**FIGURE 4:**
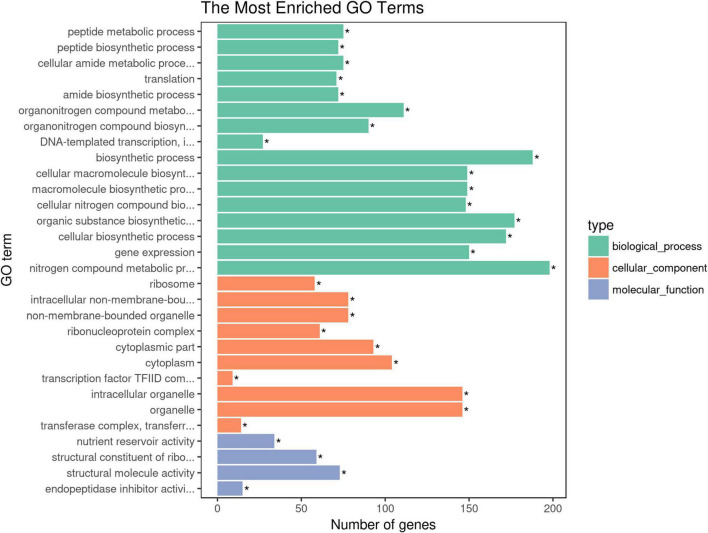
The GO terms enrichment analysis of candidate genes. The asterisk means the *P*-value is less than the significance levle of 0.01.

**FIGURE 5:**
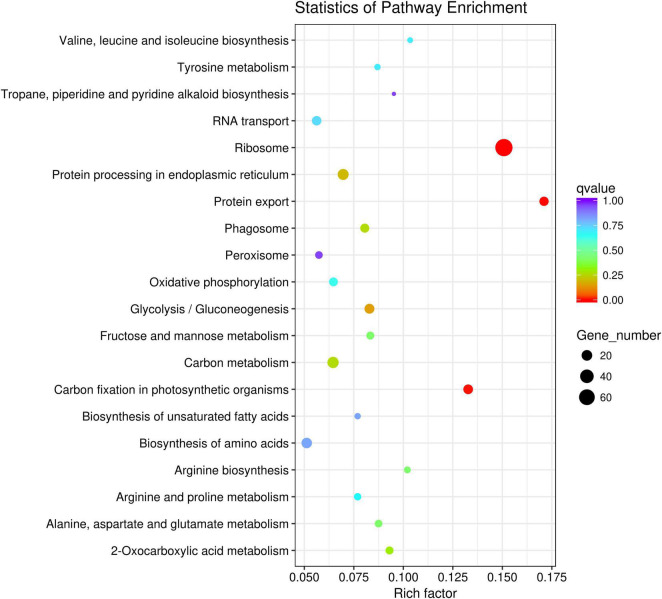
The KEGG pathway enrichment analysis of candidate genes.

Gene Ontology (GO) annotation and KEGG pathways analysis were performed to uncover the possible function of 806 candidate genes using online tools of NovoMagic (Figures 4, 5). As a result, 806 genes were annotated and categorized into three GO terms, including biological process, cellular component and molecular function. Most genes are classified into biological processes, such as nitrogen compound metabolic process, biosynthetic process, organic substance biosynthetic process, and cellular biosynthetic process. KEGG pathway analysis revealed that 806 genes mainly were enriched in several metabolic pathways. The *q* value was used to identify the enriched KEGG pathway. The results showed that most genes were obviously enriched in Ribosome, Protein export, Carbon fixation in the photosynthetic organisms, and Glycolysis/Gluconeogenesis with the level of *q* value < 0.1. In details, 76 genes were enriched in Ribosome, 13 genes were involved in Protein export, 15 genes distributed in Carbon fixation in the photosynthetic organisms, and 16 genes belonged to Glycolysis/Gluconeogenesis.

### Identification of Key Modules and Genes by Co-expression Network Analysis

To further identify the candidate genes related to the formation of floury endosperm, weighted gene co-expression network analysis (WGCNA) was performed to mine hub genes and their belonging module related to phenotype using the 806 genes. Based on the soft-thresholding power of 16, all genes were clustered into ten modules defined as ten different colors via the Dynamic Tree Cut method. The genes with similar expression patterns were categorized into one same module. For example, 48 genes were grouped into the black module, 156 genes were classified into the blue module, 110 genes were involved in the brown module, and 63 genes belonged to the green module. Otherwise, 174 genes were assigned to the turquoise module. The magenta, pink, purple, red and yellow modules contained 41, 45, 34, 55, and 66 genes.

**FIGURE 6:**
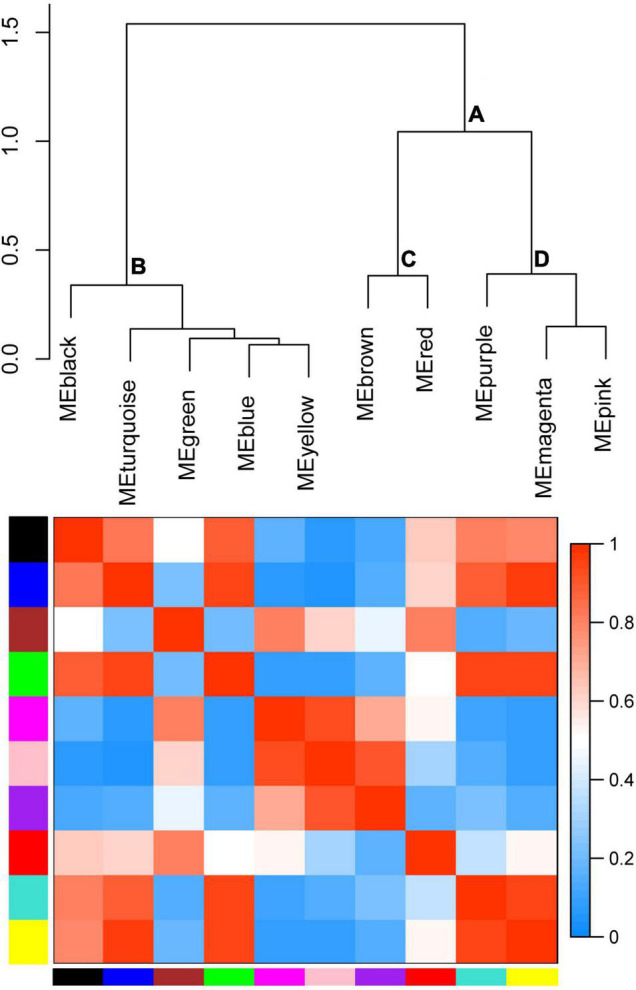
Hierarchical clustering dendrogram of module eigengenes and heatmap plot of the adjacencies. In the heatmap, green color represents low adjacency (negative correlation), while red represents high adjacency (positive correlation).

**FIGURE 7:**
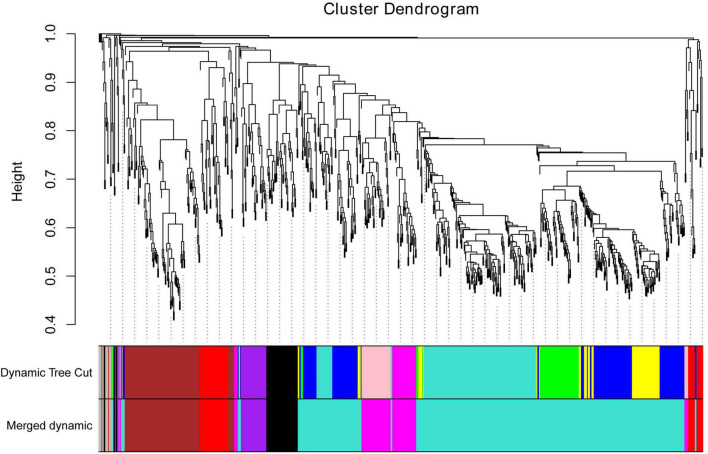
Cluster dendrogram of candidate genes, with dissimilarity based on topological overlap, together with assigned merged module colors and the original module colors. Hierarchical cluster tree of co-expression modules identified via the Dynamic Tree Cut method. The minModuleSize was 30. The MEDissThres was set as 0.2. Different colors displayed the different modules. Each leaf in the tree represented one gene. The major tree branches are originally composed of 10 modules and eventually merged into 6 modules. Each color represented one module.

To cluster module eigengenes, the similarity and dissimilarity of module eigengenes were calculated. The result showed that all modules could be divided into A and B two categories (Figure 6). A category contained C and D, two families. Red and brown modules were classified as C family. The magenta, purple and pink modules were involved in the D family. B category contained black, turquoise, green, blue and yellow modules. Based on the similarity and dissimilarity of module eigengenes, all modules were merged using the automatic merging function with the MEDissThres value of 0.2. Eventually, six modules were obtained, including black, brown, magenta, purple, red, and turquoise (Figure 7 and Supplementary Table 2).

**FIGURE 8:**
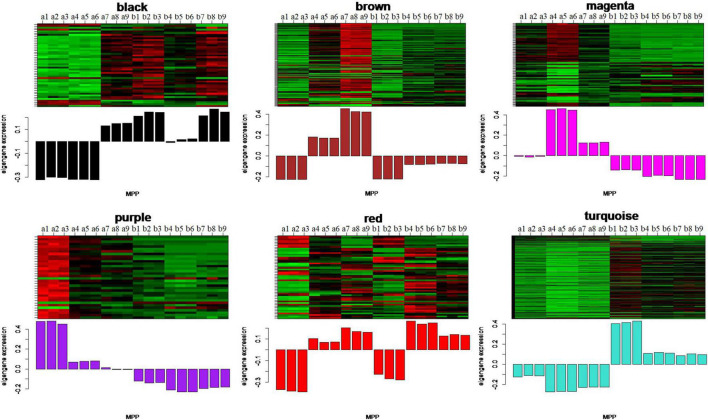
The eigengenes expression patterns and gene expression heatmap across samples for six identified modules. In heatmap, the letter a indicated W056, letter b represent W042. The numbers 1, 2, and 3 meant three biological repeats at the 20 DAP stage, 4, 5, and 6 showed three biological repeats at the 25 DAP stage, 7, 8, and 9 represented three biological repeats at the 30 DAP stage.

To identify the modules related to the structure formation of floury endosperm, the patterns of eigengenes expression and the module heatmap were displayed using the plotMat function. As is shown in Figure 8, six modules presented different expression patterns. According to the uniformity of the endosperm structure formation from 20 to 30 DAP, the purple module (55 genes) and turquoise module (459 genes) were predicted to be closely related to the formation of floury endosperm for its expression pattern. Further annotation of the genes in both modules revealed that most genes in the purple module were associated with biological processes, defense response, and cellular processes, and genes in the turquoise module were involved in organic metabolic process, organic substance transport, nitrogen compound metabolic process, and protein metabolic process. The module-trait relationship analysis further confirmed that the turquoise module is the key module related to the floury endosperm formation (Supplementary Figure 1).

**FIGURE 9:**
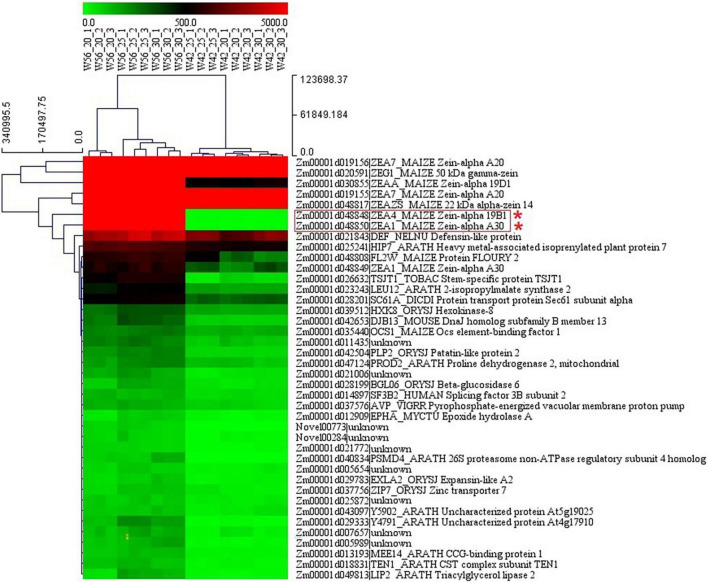
The expression pattern of candidate hub genes from different biological replicates of W056 and W042. The heatmap represents the FPKM values from RNA sequencing data.

**FIGURE 10:**
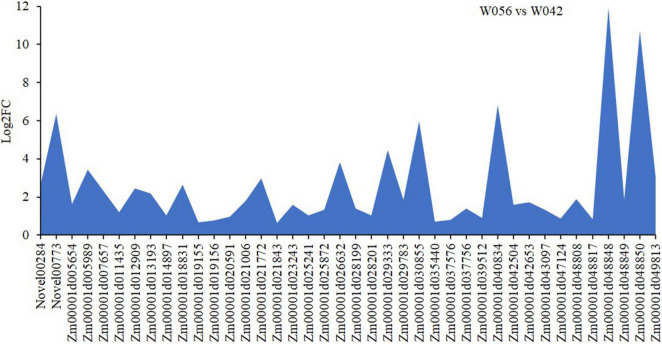
Different expression of candidate hub genes of W056 vs W042. The Log2FC values of were calculated based Q19 on the average FPKM values at 20, 25, and 30 DAP of W056 and W042.

In order to identify the hub genes, the parameters of intramodular connectivity including kTotal, kWithin, kOut, and kDiff for each gene were calculated (Supplementary Table 3). The kME value representing the correlation between the gene expression and the module eigengene was calculated too (Supplementary Table 3). Otherwise, the correlation between the gene expression and the endosperm trait were analyzed (Supplementary Table 3). As a results, 40 genes in turquoise module were isolated with the value of kME < −0.90 (Supplementary Table 3 and Figures 9, 10). Among these genes, nine zein genes including two *zein-alpha A20* (*Zm00001d019155* and *Zm00001d019156*), two *zein-alpha A30* (*Zm00001d048849* and *Zm00001d048850*), one *50 kDa gamma-zein* (*Zm00001d020591*), one 22 kDa alpha-zein 14 (*Zm00001d048817*), one *zein-alpha 19D1* (*Zm00001d030855*), one *zein-alpha 19B1* (*Zm00001d048848*), and one *FLOURY 2* (*Zm00001d048808*) were identified closely related the floury endosperm formation. In addition, both *zein-alpha 19B1* (*Zm00001d048848*) and *zein-alpha A30* (*Zm00001d048850*) presented the highest expression level in floury endosperm and the maximum values of Log2FC (W056 vs. W042), which confirmed that they were closely related to the formation of the floury endosperm of W056 (Figures 9, 10).

## Discussion

Ordinary corn kernel endosperm is composed of two parts, a horny part and a floury part. In the horny part, the starch granules are embedded compactly in the protein membrane; in the floury part, the starch granules are arranged loosely (Kikuchi et al., 1982; Gwirtz and Garcia-Casal, 2014). Generally, both parts were unevenly distributed in one endosperm with different proportions (Xu et al., 2019). Nevertheless, some special corn germplasms were created by previous researchers. The flint corn is characterized by a small soft granular center surrounded by a vitreous endosperm layer. The floury corn is identified as containing soft starch throughout the entire endosperm. Previous studies demonstrated that the starch isolated from floury endosperm is easier to gelatinize and higher in viscosity, swelling value, and α-amylase digestibility than the starch from the horny endosperm (Kikuchi et al., 1982; Gwirtz and Garcia-Casal, 2014). Proteomic analysis of the enzymes related to the starch biosynthesis with different endosperm types in maize reveals that the starch accumulation, amylose content, granule size and crystallinity percentage are different at 20 DAPS (Juárez-García et al., 2013). In the experiment, the mature seeds were used to conduct phonotype observation and biochemical analysis. Most of the results are in accord with the previous studies. For example, the floury endosperm contained higher amino acid content and had a broader molecular weight distribution. It was filled with small, loose and scattered irregular spherical shape starch granules.

**FIGURE 11:**
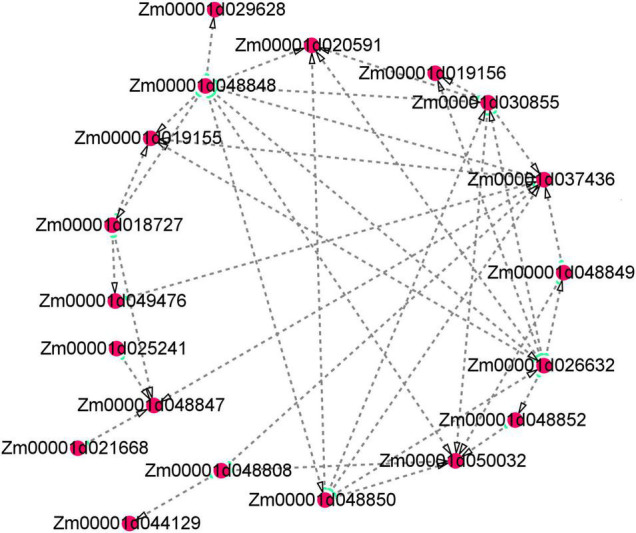
Visualization of co-expression gene network of hub genes by Cytoscape. Key hub genes identified by WGCNA indicated by larger and green circles.

Several floury genes, including *fl1*, *fl2*, *fl3*, and *fl4*, were cloned (Coleman et al., 1997; Holding et al., 2007; Wang et al., 2014, 2018). In this study, an *fl2* gene (Zm00001d048808) was identified. The expression pattern analysis showed that the gene displayed a higher expression level in the floury endosperm of W056. A previous study revealed that *fl2* mutation exhibited smaller, asymmetrical and misshapen protein bodies (Coleman et al., 1997). The expression of the *fl2* gene in transgenic maize triggered the accumulation of the 24-kDa a-zein protein (Coleman et al., 1997). In the experiment, gene network analysis showed that the *fl2* gene act on the expression regulation of *Zm00001d044129*, *Zm00001d037436*, and *Zm00001d050032* (Figure 11). Among three genes, *Zm00001d044129* encodes a glucose-1-phosphate adenylyltransferase large subunit 1, *Zm00001d050032* encodes a glucose-1-phosphate adenylyltransferase small subunit 2, and *Zm00001d037436* is an unknown gene. The Zein proteins have mutual interactions (Kim et al., 2002). For example, α-zeins and δ-zeins have strong interactions. The 16-kD γ-zeins and 15-kD β-zeins closely interacted with each other. The domains within the 22-kD α-zeins bounded preferentially not only the α-zeins and β-zeins but also the β-zeins and γ-zeins. Otherwise, maize opaque2 (O2) could regulate *27-kD*γ*-zein* gene expression (Zhang et al., 2015). In this study, two genes, *zein-alpha 19B1* (*Zm00001d048848*) and *zein-alpha A30* (*Zm00001d048850*), were found almost exclusively expressed in the floury endosperm of inbred line W056. Their expression level and Log2FC values of W056 vs. W042 were far more than that of other genes (Figures 9, 10). Both genes function as source genes in the expression regulation of other genes (Figure 11).

## Conclusion

In this study, floury endosperm corn was identified in contrast to normal flint corn. The corn with floury endosperm was filled with the small, loose and scattered irregular spherical shape starch granules and contained higher amino acid content. Dynamically comparative transcriptome analysis combined with weighted gene co-expression network analysis of endosperm at 20, 25, and 30 DAP, a total of 806 genes and six modules were identified. And the turquoise module with 459 genes was proved to be the key module closely related to the floury endosperm formation. Nine zein genes in turquoise module were identified closely related the floury endosperm formation. Both *zein-alpha 19B1* (*Zm00001d048848*) and *zein-alpha A30* (*Zm00001d048850*) function as source genes with the highest expression level in floury endosperm and the maximum values of Log2FC (W056 vs. W042).

## Data Availability Statement

The datasets presented in this study can be found in online repositories. The names of the repository/repositories and accession number(s) can be found in the article/Supplementary Material.

## Author Contributions

TL and CL designed the experiment. TL performed the experiments, generated figures, and wrote the draft manuscript. FK and QD helped with transcriptome analysis and revision of the manuscript. DX and YL helped in performing experiments. GL, HY, and YZ were responsible for the cultivation of waxy corns and prepared the material. All authors have read and approved the final manuscript.

## Conflict of Interest

The authors declare that the research was conducted in the absence of any commercial or financial relationships that could be construed as a potential conflict of interest.

## Publisher’s Note

All claims expressed in this article are solely those of the authors and do not necessarily represent those of their affiliated organizations, or those of the publisher, the editors and the reviewers. Any product that may be evaluated in this article, or claim that may be made by its manufacturer, is not guaranteed or endorsed by the publisher.

## Footnotes

1
http://www.novogene.cn/

2
ftp://ftp.ncbi.nlm.nih.gov/genomes/genbank/plant/Zea_mays/latest_assembly_versions/GCA_000005005.6_B73_RefGen_v4

3
https://www.ncbi.nlm.nih.gov/bioproject/PRJNA820289

4
https://magic.novogene.com

## Supplementary Material

Supplementary Figure 1 The module-trait relationship between W056 and W042.

Supplementary Table 1 All genes identified by transcriptome analysis.

Supplementary Table 2 The list of 806 candidate genes and their belonging module.

Supplementary Table 3 The intramodular connectivity, module membership, and the correlation with trait for each gene.
