# Supplementary figures and images for: Dynamic Transcriptome-Based Weighted Gene Co-expression Network Analysis Reveals Key Modules and Hub Genes Associated With the Structure and Nutrient Formation of Endosperm for Wax Corn
Source: Front Plant Sci. 2022 Jun 9;13:915400. doi: 10.3389/fpls.2022.915400 (PMC9218491; doi:10.3389/fpls.2022.915400)

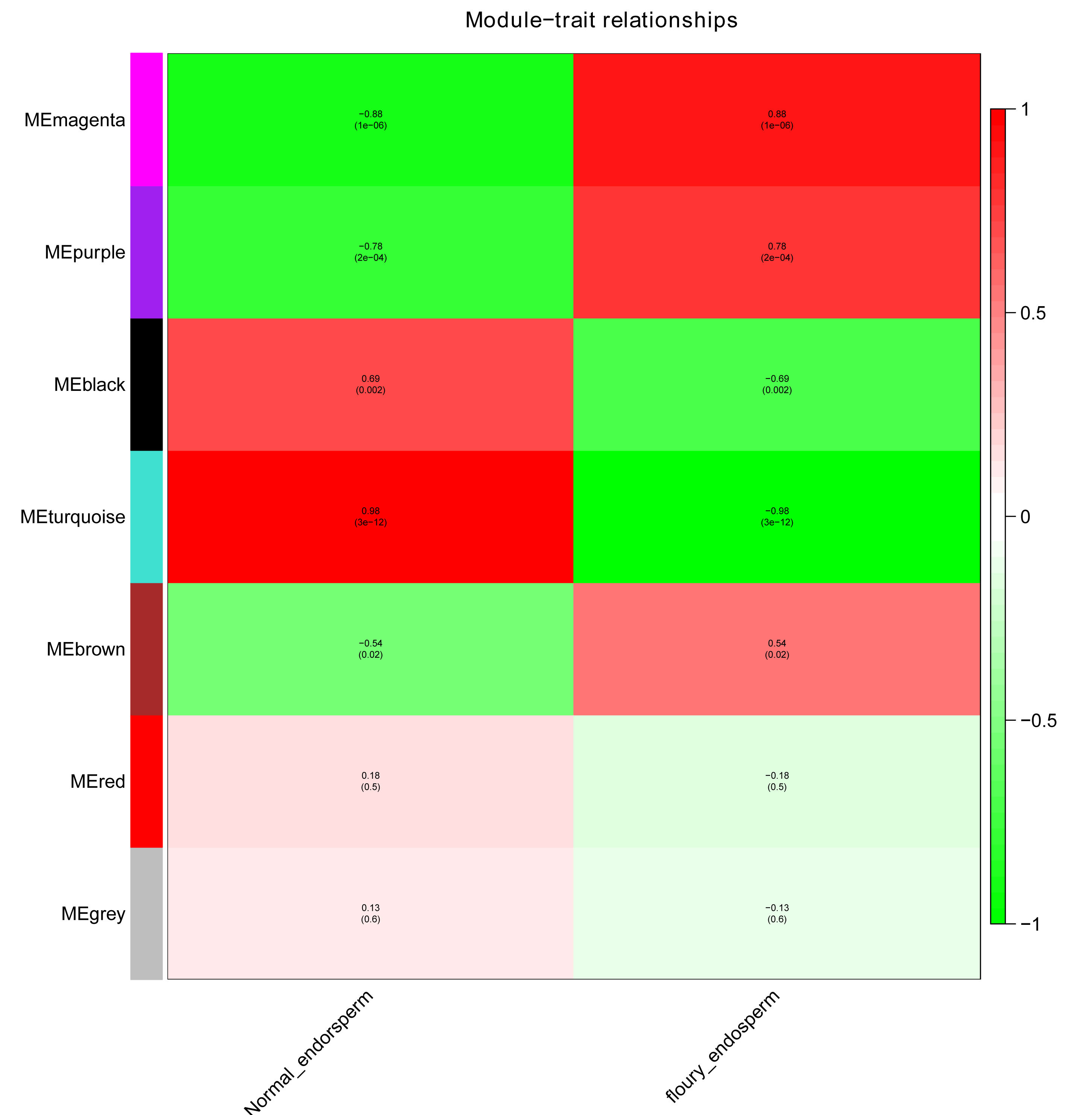

Supplement: Supplementary Figure 1 — The module-trait relationship between W056 and W042. [file Image_1.JPEG]
